# A new predictive coding model for a more comprehensive account of delusions

**DOI:** 10.1016/S2215-0366(23)00411-X

**Published:** 2024-01-16

**Authors:** J Harding, N Wolpe, SP Brugger, V Navarro, C Teufel, PC Fletcher

**Affiliations:** 1 School of Clinical Medicine, University of Cambridge, Cambridge CB2 0SZ, UK; 2 Department of Psychiatry, University of Cambridge, Cambridge CB2 0SZ, UK; 3 Department of Physical Therapy, The Stanley Stever School of Health Professions, Faculty of Medicine, Tel Aviv University, Tel Aviv 6997801, Israel; 4 Sagol School of Neuroscience, Tel Aviv University, Tel Aviv 6997801, Israel; 5 Cardiff University Brain Research Imaging Centre (CUBRIC), School of Psychology, Cardiff University; 6 Centre for Academic Mental Health, Bristol Medical school, University of Bristol; 7 Cambridgeshire and Peterborough NHS Foundation Trust, Elizabeth House, Fulbourn, Cambridge CB21 5EF, UK; 8 Wellcome Trust MRC Institute of Metabolic Science, University of Cambridge, Cambridge Biomedical Campus, Cambridge CB2 0QQ, UK

## Abstract

Attempts to understand the unshared reality of psychosis raise questions about how the brain models the world. Standard predictive coding approaches suggest that it does so by minimising mismatches between incoming sensory evidence and predictions. By adjusting predictions, we converge iteratively on a “best-guess” of the nature of the reality. Recently, it has been argued that a modified version of this framework − hybrid predictive coding − provides a better model of how healthy agents make inferences about external reality. We suggest that this more comprehensive model furnishes us with a richer understanding of psychosis. In this personal view, we briefly describe the hybrid predictive coding model and show how it offers a more comprehensive account of the phenomenology of delusions, thus providing a potentially powerful new framework for computational psychiatric approaches to psychosis. We also make suggestions for future work that will be important in formalising this novel perspective.

## Introduction

Computational psychiatry shares the aspirations of cognitive neuropsychology^1^ and neuropsychiatry^2^: to scrutinise and understand symptoms of mental illness in the context of the functional attributes of the healthy systems that have been altered or perturbed.^3^ Predictive coding models of psychiatric symptoms have played a significant role in computational psychiatry, promising a principled description of how relatively simple disturbances in the brain’s computations can account for an array of complex experiences.^4,5^ These models offer a genuine hope for bridging the explanatory gap between neurobiological, cognitive, and subjective levels of explanation, and, ultimately, for a comprehensive multilevel perspective on the causes, emergence, and course of the symptoms of many psychiatric conditions.^6–9^ This approach has been especially useful in our attempts to understand psychosis: a complex experience of an alternative or unshared reality, characterised by delusions (seemingly irrational yet firmly held beliefs) and hallucinations (perceptions occurring in the absence of causative stimuli). By applying a model of healthy inference in the brain to explore psychosis, predictive coding models, it has been claimed, can account for both types of experience.^4^

The standard predictive coding framework suggests that the brain models reality through an iterative process, wherein top-down beliefs are adjusted to minimise prediction errors between incoming sensory evidence and the agent’s predictions of this evidence. However, it has been argued that a modified version of this framework − hybrid predictive coding^10^ − provides a better model of how healthy agents make inferences about external reality. It follows that this more comprehensive model may furnish us with a more complete apprehension of the nature of psychosis, one which ultimately allows us to relate delusions and hallucinations to underlying neurobiology.

Explanations of delusions might benefit particularly from what the hybrid predictive coding model has to offer. Whilst the prodromal phase of psychosis and the initial emergence of delusional beliefs has been compellingly framed in terms of alterations in the iterative inference processes outlined by standard predictive coding models (discussed below), there are several core aspects of established delusions, prominent in clinical practice, that do not readily lend themselves to such an account. For example, a delusional belief may appear suddenly as a revelatory, self-evident, all-explaining, and unshakeable insight that seems impervious to contradictory evidence. The suddenness with which delusional beliefs may arise is hard to reconcile with the process of iteratively-refined inferences suggested by predictive coding. The standard predictive coding model struggles to account for the transition from the unstable prodromal phase to the stable delusional state, and for the fixedness of established delusional beliefs. How do we explain the sometimes very definite point at which a person moves from a search for explanations into a strong conviction that they have found the over-arching truth? Current models of delusions would be more valuable for both research and clinical practice if they were better able to provide mechanistic explanations for such key phenomenological features. This would also offer greater potential for inspiring treatments.

The hybrid predictive coding framework expands standard predictive coding by adding an amortisation component that provides the top-down process with an initial best guess from which to start the iterative process. This initial best guess is a rapid, gist-based route to inference, learned through experience. As such, it can offer speed and efficiency in stable environments. But it may fail where circumstances are more volatile or changeable. We note that the amortised/iterative inference dichotomy may be fruitfully applied to accounts of delusions that go beyond predictive coding, but which fall within the wider field of modelling cognition and behaviour as arising from (approximately) Bayesian inference.^11–13^ However, we focus particularly on predictive coding due to its substantial influence on recent theorising about delusions, as well as wider psychotic phenomena. We begin by briefly reviewing existing predictive coding accounts of delusions, highlighting their explanatory limitations. We then move on to discuss the hybrid account and show how the addition of amortised inference provides a richer perspective on delusions, one that more comprehensively addresses the phenomenological aspects that have hitherto been neglected.

### Predictive coding and delusions

The hierarchical predictive coding model posits that, since the brain cannot directly access the external world, it infers the most likely cause of an experienced sensory input by using a hierarchically-organised set of predictions.^14–20^ This can be formalised in Bayesian terms: a probabilistic prediction (prior) is combined with the sensory data (likelihood) to compute the most likely cause (posterior). Prediction error signals resulting from this process indicate that the brain’s current inference about the world is inaccurate and may need updating. By iteratively updating prior beliefs to minimise prediction errors, the brain more accurately perceives and models the external world. This updating occurs both quickly, as part of the perceptual process in each interaction between the agent and its environment, and more slowly, as an updating of the agent’s generative model that occurs over many instances of such interactions. Estimated precision of prediction errors at different levels determines the extent to which priors are updated; the more precise the sensory data is estimated to be relative to the prior, the more the sensory-level prediction error will drive updating of the prior.

When applied to delusions, the predictive coding framework appeals to an imbalance in the integration of priors and evidence. The core atypicality is formulated as an increase in the estimated precision of the sensory evidence relative to the precision of the prior, causing the brain’s model of reality to become perturbed through relying unduly on sensory inputs (rather than pre-existing knowledge of the world).^4,14,21–24^ This predictive coding model has provided compelling explanations for several features of delusions, such as the prodromal period,^25^ in which there is excessive uncertainty, a sense of altered salience and a feeling that new beliefs (hypotheses) must be generated to encompass unusual experiences. Experientially, the relatively reduced precision of prior beliefs (compared to that of incoming sensory data) would lead to a sense of uncertainty and change: a powerful feeling that one’s existing world-model was no longer valid and that there was something that needed to be explained. Patterns and coincidences would become salient and everyday occurrences might feel pregnant with meaning. Thus, a relatively low-level perturbation in the process of Bayesian inference can be related to the delusional mood or “Wahnstimmung”^26^ that characterises the phenomenology of the prodromal period of psychosis.

Through its explanation of the prodromal phase, the predictive coding approach effectively offers computational explanations for subjective experiences. There has been much drive in recent psychiatric research to unite contemporary neuroscientific approaches to delusions with the phenomenological perspective.^27,28^ Indeed, a recent editorial in *The Lancet Psychiatry*^29^ highlights the importance of a phenomenological approach in potentially bridging divisions between patients and professionals. It is in the attempt to bridge this explanatory gap − a quest for consilience^30^ − that computational psychiatry could offer great potential.^31–35^

However, despite real progress in explaining some of the phenomenology of psychosis in computational terms, there remain several key experiential features of delusions that cannot be so neatly explained by the predictive coding model. One key example is the model’s difficulty in readily explaining how the stable and entrenched delusion − a new set of beliefs that satisfies the quest for understanding and resolves the many puzzles of the prodromal phase − emerges from the state of profound uncertainty, flexibility, and explanation-seeking characteristic of the delusional mood. Indeed, by its very nature, a predictive coding process emphasises that contradictory evidence is the drive to updating. So how might the same model explain fixed, impervious beliefs? Some suggestions appeal to a distinction between high- and low-level priors, with the former gaining additional weighting (or precision) as an adaptive response to noisy evidence.^4,36^ In other cases, a related argument is that entrenchment is a consequence of a persistent failure of the model, despite repeated updating, to make error-free predictions.^37,38^ That is, repeated failures of predictions lead to a reduced learning rate and a growing tendency to ignore new evidence.^39^ These suggestions, however, fail to explain the crucial shift in which a “hunger” for new priors (in the prodromal phase) is satisfied by a new belief system that is rapidly established and soon unassailable (as in the delusional state). Moreover, such added-on explanations may call for caution insofar as they highlight the poorly-specified nature of a framework that seems able to accommodate all possibilities.^40^

In addition to its difficulty in accounting for the point of shift from the prodrome to the delusional state, and the fixed beliefs of the delusional state itself, the predictive coding account struggles to explain the so-called ‘insight experiences’ reported in clinical practice. Jenson, writing about his own experiences of schizophrenia, reports that “believing that one is influenced by an alien force does not have the experiential quality of a reasoned conclusion. Rather […] there is an idea revealing itself, an idea that has its own or is its own stimulus. It carries a sense of truth and certainty; it is what it is by nature of itself. This idea is automatous and expresses a higher truth that supersedes one’s own thoughts or knowledge about the ordinary world”.^41^ The relatively sudden and fully-formed presentation of some delusional beliefs, accompanied by this sense of indubitability and revelation, is difficult to reconcile with the core idea of predictive coding: an iterative process of error detection and updating.

In short, the standard predictive coding framework has promise but, in the face of a more comprehensive phenomenology of delusions, its shortcomings become apparent. Most importantly, existing predictive coding models fail to adequately explain the transition from the prodromal to the delusional state, in which the quest to update is replaced by a fixed belief, and are unable to capture situations in which delusions rapidly emerge as sudden and unassailable revelations that seem to pervade the person’s entire view of the world and its possibilities. Below, we describe a more recent extension of the predictive coding framework and suggest ways in which it can solve these problems.

### The hybrid predictive coding model

The hybrid predictive coding model proposes that, alongside the cycle of inference and updating central to standard predictive coding, there is a system of ‘amortised inference’ that learns a direct mapping between inputs and beliefs to streamline the process of inference.^10^ That is, rather than starting the iterative process from a belief picked at random, the iterative process instead begins from a belief at which previous iterative processes arrived under similar conditions.

To illustrate, consider the following problem: if 2*x* = 20, what is *x*? Assuming a basic understanding of algebra, you will probably have answered *x* = 10. What would you conclude if we attempt to convince you that *x* = 0? Probably that we need to brush up on our arithmetic. However, this is a perfectly rational answer. Why? Because if we substitute *x* for 0, we produce 20 = 20. Both the rapid arrival at the answer *x* = 10 and the subsequent difficulty in understanding the logic behind concluding *x* = 0 (until it is explained) illustrate amortised inference. The learning of stable contingencies between inputs and their (inferred) causes enables rapid employment of the previously-taught inference and prohibits iterative analysis of the conclusion settled upon. Note how the assumption that the problem was an *algebraic* one was made without reflection. However, once this algebraic context has been established, it sets limits on the space of possibilities that one entertains in reflecting on the problem; the subsequent inference becomes inflexible and cannot be overridden easily. In this sense, amortised inference can be compared to the acquisition of a ‘mental habit’.^42^

According to the hybrid model, amortised inference maps the input to an initial belief state − the optimal starting inference or ‘best guess’ − which is then refined by the subsequent iterative process via standard prediction error minimisation. Any prediction error between the amortised prediction and the refined posterior − indicating an inaccurate first guess − is used to adjust the parameters of the amortised function. Therefore, after each completed inference, the amortised component tries to learn a mapping from the input to the inference that the iterative component has settled on. The better this mapping is, the less there is a need for refinement by the iterative component during the next interaction between the agent and the environment. Once an accurate mapping has been learned, inference can be achieved nearly instantaneously after a single feedforward sweep, without iterative refinement, thus minimising computational cost.

However, whilst amortised inference is efficient and rapid in stable environments, when data are sparse or when the environment is unstable the inference is vulnerable to inaccuracies.^43^ As such, environments in which input-cause contingencies can be learned reliably provide the opportunity for rapid and computationally cheap inference, whereas more dynamic and unpredictable environments require a shift towards the iterative approach which allows greater accuracy, but with additional computational cost. The hybrid model is inherently sensitive to how much it needs to rely on each component: a sub-optimal initial best guess offered by the amortised component will generate prediction errors, which will call for refinement of this initial guess by the iterative component, thereby upregulating iterative inference. ^10^

Given that the iterative component uses prior beliefs to predict sensory inputs, it is inherently sensitive to one’s understanding of the current state of the world. By contrast, the amortised component ignores the question of whether a certain state of the world is likely per se, and simply establishes how likely an inferred state is to have caused the current input. This notion partially resembles the distinction made by Teufel and Fletcher^18^ between two forms of prediction; one relating to prior knowledge of local, context-specific regularities − so-called “expectations” − and the other to knowledge of global, context-independent regularities − referred to as “constraints”. In some ways, constraints are analogous to cached predictions used in amortised inference: applied ineluctably and inflexibly allowing for rapid, efficient inference but prone to error, particularly in unstable environments. This also invites consideration of amortised inferences, like constraints, as bottom-up, in the sense that they are applied as a function of data rather than higher level beliefs.

To summarise, the hybrid predictive coding model suggests that belief formation is optimised by a joint system of fast and slow inference: ‘assumptive’ amortised inferences dominate when the agent can capitalise on stable regularities, with a corrective iterative system evaluating these inferences against the refined integration of prior beliefs and sensory evidence. Amortised inference may be considered akin to ‘habitual’ coding insofar as it reflects a learned mapping, obviating the need to inferentially “reinvent the wheel”.^44^ The initial inferences generated by this mapping are then subject to the iterative inferential processes characteristic of the standard predictive coding model and the ultimate output of this complementary inferential process creates a mapping that is used for future amortised inference.

### A hybrid predictive coding model of delusions

We now explore how the hybrid model may allow a fuller account of the phenomenology of delusions than that provided by the standard predictive coding model. It is important to note that hybrid predictive coding was conceptualised as a general model of inference, and that delusions have not yet been formally explored within this model. Nevertheless, we can consider two components of amortised inference which, if pathological, might contribute to the emergence of delusions: first, the selection and application of the appropriate mapping function for amortised inference and, second, the interaction between the amortised prediction and the iterative process. It is important to acknowledge that our proposal for how hybrid predictive coding might contribute to our understanding of delusions remains preliminary and conceptual. We speculate on possible directions of future development in the panel.

First, we consider how the choice of mapping function might contribute to delusion formation. The hybrid model posits that previously reliable environmental regularities drive rapid, system-wide predictions via a learned feedforward mapping from specific sensory inputs to a hierarchical series of inferences. Although not part of the current hybrid predictive coding model, this idea suggests that we might need to develop a ‘library’ of possible amortised input-cause mappings based upon past successful inferences.

The success of amortised inference depends on the selection of the correct mapping in the correct context. If we are watching a horror film and a drawer is opened to reveal a bread knife, the inferences we make will be markedly different from those accompanying a similar perceptual experience when watching a cooking programme or at the home of a friend. In the context of the horror film, we ‘just know’ that the bread knife will play a sinister part in future events. The amortised chain of inferences proceeds unrectified by iterative analysis because our prior beliefs about ominous symbolism in horror films encompasses such a conclusion. In delusions, a perturbation in the system that selects the mapping from the library might enable the same conclusion in an incorrect context. That is, the person might, automatically and unquestioningly, infer ominous consequences seeing a knife in the home of a friend.

What might underpin this ‘wrong’ choice of mapping? We discussed above how the pathological iterative process proposed by the standard predictive coding model could give rise to the prodromal sense of uncertainty and search for understanding. Sips describes, from his personal experience of psychosis, how this delusional mood “makes one literally question everything”.^45^ If this reflects a highly uncertain (imprecise) set of priors, the iterative process will blindly follow a path towards beliefs that perfectly explain incoming sensory evidence, disregarding the beliefs built through previous experience. Weak priors serve a purpose in increasing the sensitivity to new contingencies but, if excessively so, one’s past experience becomes an unreliable benchmark, conferring an increased tendency to accept an initial best guess generated by the amortised component. For instance, an incorrectly selected amortised mapping that attaches an ‘ominous’ conceptual inference to the perceptual inference of ‘bread knife’ is less likely to be corrected if the prior belief − that, say, knives in a friend’s kitchen are benign - becomes uncertain. Note that, in this instance, it is not the case that there is something wrong with the process or mechanisms of amortised inference, per se, but rather that the abnormal precision weighting could result in a ‘false’ mapping (again, not necessarily one that is inherently abnormal) that persists uncorrected. Thus, the existence of a perturbation of the process of iterative inference captured by standard hierarchical predictive coding − a perturbation hypothesised in previous accounts of delusions^4,14,22,46–52^ − could open the door for an amortised inference that would, under other circumstances, be corrected.

Furthermore, the library of mappings will vary across people, making the individual’s personal experiences and preoccupations very relevant in considering the mappings selected. It seems credible that past trauma experience could play a major role in creating this library and could perhaps make the selection of a mapping to a more paranoid inference less likely to result in prediction error with the iterative component. This is in line with evidence that childhood trauma is associated with the development of hallucinations and delusions in psychotic disorders,^53–55^ perhaps by a resultant ‘pessimistic’ worldview increasing the probability of iteratively inferring a negative experience from neutral or ambiguous sensory evidence.^56^ In this case, not only is selection to an ‘incorrect’ mapping more likely, but the change in worldview (in one’s generative model) makes this incorrect mapping less likely to be rectified by iterative inference.

Delusional beliefs are more likely to be found at higher levels of the hierarchy (the more abstract contextual inference) than at lower levels (the perceptual inference). The higher up the hierarchy, the slower the timescales on which iterative processes operate. At lower hierarchical levels, iterative mechanisms operate rapidly but at higher cognitive levels, iterative timescales lengthen.^57–59^ The timescale of amortised processing, on the other hand, is plausibly independent of hierarchical level. Therefore, amortised mappings could conceivably perform high-level, conceptual inferences with perception-like levels of speed. This idea of using purely an amortised mapping that is rapidly and reflexively applied across a hierarchy of inferences could explain the profound, seemingly all-explaining nature of a range of delusional experiences. Phenomenologically, delusional beliefs are perception-like; they are both beliefs about the world but are also experiences of the world that are self-evident. Even non-delusional beliefs sometimes have this quality. Consider arriving home to a smashed front door and scattered belongings; the immediate amortised mapping to ‘my house has been robbed’ is more than a simple *belief*; it is experienced as a fact of the world in the same way as the visual perception of the damage. The addition of an amortised encoder to predictive coding models offers a new explanation of this phenomenological quality of delusional beliefs by considering the characteristics of the amortised mapping.

In our first point, we have argued that selection and application of an incorrect mapping could contribute to delusion formation and underpin the perception-like phenomenological quality of delusional beliefs. Next, we focus on a second possibility relating to how the interaction between the amortised and iterative components may underlie delusional thinking. Specifically, we suggest a mechanism by which uncertain iterative inference, as experienced in the prodromal period, can transition into an incorrect amortised inference that fails to be corrected.

All people make rapid and error-prone inferences, and it is the role of the ensuing iterative inference process to rectify these. Recall that the iterative process operates via progressive minimisation of prediction error - a process operationalised in computational modelling as ‘gradient descent’, where the goal is to find the inference for which prediction error (the ‘loss’) is at a minimum. To illustrate, imagine you are standing at the top of a valley in the dark. To reach the bottom, you keep taking steps in the direction of the steepest descent, making small adjustments at each step until the gradient is zero. Although in reaching this point you might assume you had reached the bottom of the valley, this point might not be the deepest place in the valley; it could just be a pit in the hillside. Whilst there is one ‘global minimum’ (overall ‘correct’ inference) for every sensory input, there may be many ‘local minima’ - points which minimise prediction errors *only up to a certain level*. The deeper and more numerous the pits, the more hidden the true global minimum will be. Therefore, highly elastic evidence that fits with many different inferences will make arriving at the correct hierarchical inference very difficult.

Consider social inferences. Specifically, the act of inferring the attitude of other people towards oneself − a process which appears to go awry frequently in certain conditions associated with delusional ideation. The evidence (subtle social cues) upon which such inferences are based tends to be weak or and/or open to multiple interpretations, meaning that inferences may naturally be guided by other, less strictly relevant but more strongly held prior beliefs.^60^ Suppose, for example, that you make a mistake during a presentation to a crowd. Is the smile of your colleague meant to indicate friendly support, or are they gloating? Interpreting the smile as gloating rather than friendly may represent one of these local minima and may persist because further iterations will not generate sufficient evidence to escape from this pit. Therefore, whilst iterative inference can minimise prediction error, this is no guarantee that the individual will settle on the appropriate inference, partly because the initial best guess that is provided by the amortised component may adequately account for the immediate sensory evidence (that the person is smiling) even if the higher inference (that the person is smiling *gloatingly*) is erroneous.

Importantly, if the initial best guess falls close to a local minimum, we can arrive (via iterative inference) at a region with gradient zero which is not necessarily the optimal inference, but which will be difficult to escape from. Both the standard and hybrid predictive coding models could arrive at local minima. However, a crucial difference may emerge under conditions of uncertainty, such as in the prodromal phase, where priors might be uncertain as we discussed above. Due to environmental variability, a purely iteration-based predictive coding algorithm will arrive at relatively imprecise priors and will therefore tend towards a random starting point. Amortised inference, on the other hand, will find a starting point based on a previously-learned mapping, which will more likely be close to a minimum. Specifically, if the iterative component erroneously settles on a final inference that is based on a local minimum, the amortised component will be updated in line with this erroneous inference. In the next interaction between agent and environment, the initial best guess of the amortised component will therefore be closer to this local minimum, causing the amortised component to be updated in line with the incorrect inference again, and so on. If this process repeats over days and weeks, amortised initialisation could cause the iterative component to become stuck in a local minimum, even if the iterative refinement of the initial best guess is operating normally, in the same way that once your colleague’s smile has been interpreted as gloating, there is an increased tendency to interpret future actions of that person as carrying malicious intent. This effect might be particularly pernicious where the evidence is noisy, such as in the social domain.

We believe that it is a fascinating possibility that the process we just described could underpin the phenomenological shift from the delusional mood into the stable and entrenched delusional state. Iterative inference producing high levels of uncertainty is replaced by amortised inference that maps directly to a (false) local minimum, one that has the potential to cause a shift in our generative model of the world. In subjective terms, this new amortised inference, which sets expectations at multiple levels of the hierarchy, might be experienced as a flash of ineluctable insight against a background of puzzlement and uncertainty.

Of course, an important consideration, leaving aside general questions about the falsifiability of predictive coding,^61^ concerns potential empirical consequences of our proposal. As indices of amortised and iterative inference respectively, we might expect learning of an amortised encoder to lead to a relative increase feedforward compared to feedback processing. Recent ultra-high field fMRI work is compatible with this, demonstrating increased layer-specific feedforward connectivity following perceptual learning.^62^ Conversely, disrupting feedback processing provides a means to experimentally manipulate the iterative component, utilising, for example, a precisely timed masking paradigm^63^ or via pharmacological means.^64^ We might expect a differential effect on performance: contexts where a mapping had previously been learned would be unimpaired relative to novel settings, revealing the operation of amortised inference. Alternatively, disruption of iterative inference during learning should prevent the establishment of an amortised mapping, leading to changes in performance once disruption ceases.

Our account would predict that delusions or delusion-like beliefs are associated with a greater increase in feedforward signalling following learning or disruption of top-down processing, sitting well with existing literature on the association with a reduced influence of prior beliefs during perception.^65^ We might furthermore expect to observe a greater degree of generalisation of this feedforward mode of processing, to untrained stimuli or contexts, which may prove advantageous in certain settings.^66^

### Concluding Section

The complex phenomenology of delusional experiences poses challenges to those who wish to understand the mechanisms by which they arise. Computational models offer opportunities to meet these challenges by bridging the gaps between the subjective experiences and the underlying cognitive and neurobiological processes. In this respect, predictive processing models have shown promise, particularly in accounting for the early phases of delusional emergence.^36^ Our aim in presenting the current ideas was to highlight what we see as certain limitations in standard prediction-based models: notably, they do remarkably well in providing a compelling account of the background state against which delusions arise as well as the content of delusional thinking once it is established. But they leave core features unexplained. Delusions go beyond flawed inference. They are perspectives on reality that may emerge rapidly and persist despite a range of evidence that they are untrue. Moreover, they carry a sense of deep insight, ultimately shaping the entire worldview. We see an important limitation in the standard predictive coding, which, though it accounts very well for a drive to updating, doesn’t seem to explain why the delusions ultimately form when they do, in the way that they do and with the remarkable incorrigibility that characterises them. We suggest that, in appealing to amortised as well as iterative inferences, hybrid predictive coding moves us further towards a comprehensive account of delusional phenomenology. The multi-level and instantaneous setting of priors by the feedforward amortised component leads to inferences that are both sudden and all-pervasive, and that confer a sense of deep insight and conviction. We see this as a crucial complement to the account provided by standard predictive coding.

### Panel Future Directions

- Formal computational modelling focusing on (i) learning and optimal selection of the amortised mapping, and (ii) how the amortised and iterative parts of the system interact (for example, whether the amortised encoder could generate training data for the iterative ‘generative model’).
- How does amortised inference − perhaps in interaction with iterative inferences − take uncertainty into account? Generalised predictive coding models iteratively estimate precision parameters.^67^ A *generalised* hybrid predictive coding model could amortise the estimation of the precision of sensory data, in addition to the predictions themselves (indeed, this is how a variational autoencoder learns a generative model of training data^68^).
- Might fundamental alterations in inbuilt ‘constraints’^16^ affect amortised precision estimation, leading to altered estimations of environmental volatility? For example, could specific changes in neural systems (e.g. noradrenergic) be identified that create an automatic bias in amortised precision or uncertainty estimation?
- Despite growing information on the neural circuitry underlying standard predictive coding,^5,69^ we currently know little about the neural underpinnings of amortised inference, or about how the interaction between the two components is modulated.
- Observing the effects of various pharmacological perturbations on predictive coding circuity, with the addition an amortised component, could contribute to our understanding of how psychotomimetic drugs may act.^44^
- As with other developments of standard predictive coding, we must determine whether and how newer models truly supersede existing approaches in their distinctiveness, validity and scope. A failure to address such questions has led to serious criticisms.^70,71^ We should focus not only on what the hybrid model adds to predictive coding accounts but also on its relationship to related but alternative accounts of psychosis.^72,73^
- Given that a more comprehensive model of healthy functioning could enrich our attempts to account for specific perturbations, the addition of an amortised component may enhance models of other psychiatric conditions such as anxiety and depression^7–11^
